# Supplementary material for: Bioinformatics analysis identifies DYNC1I1 as prognosis marker in male patients with liver hepatocellular carcinoma
Source: PLoS One. 2021 Oct 22;16(10):e0258797. doi: 10.1371/journal.pone.0258797 (PMC8535175; doi:10.1371/journal.pone.0258797)
Supplement: Attachment — Submitted filename: Response to Reviewers.doc [file pone.0258797.s011.doc]

**Response Letter**

Dear Editor-in-Chief,

Thank you very much for your letter and advice. We have revised the manuscript, and would like to re-submit it for your consideration. We have addressed the comments raised by the reviewers and editor, and the amendments are highlighted in red in the revised manuscript. The responses to the editor' and reviewers' comments are listed below this letter.

We hope that the revised version of the manuscript is now acceptable for publication in your journal.

Looking forward to hearing from you soon.

With kind regards,

Yours sincerely,

Yan Liu Dr.

Puren Hospital Affiliated to Wuhan University of Science and Technology, Wuhan, Hubei, 430081, China.

Tel/Fax: 027-86367776

Email: [liuyan@wust.edu.cn](mailto:liuyan@wust.edu.cn)

We would like to express our sincere thanks to the editor and reviewers for the constructive and positive comments.

**Replies to Editor**

In the analysis it is stated that 371 samples of LIHC and adjacent normal tissue are used. However, the total number of "normal" samples is 50, so I would like to know if in a paired analysis (among the 50 paired samples) DYNC1I1 still appears as a prominent gene.

Answer:

Thank you very much for your question. We extracted 50 pairs of matched patients and analyzed the data. The results show that DYNC1I1 is still a significant prominent gene.

On the other hand, the text included in the differential expression analysis is not very detailed. Has any kind of filter been done to eliminate low expressed genes ? with what cpm value ? The P value is adjusted ? with which method ?

Answer:

Thank you for your question. We use the R package "Deseq2" to process the counts data of the transcriptome, and use the code "dds <- dds[rowSums(counts(dds))>1,]" to delete low-expressed genes. The CPM value should be applied to the "edgeR" package, but this R package was not used in our analysis. We use the R package "Deseq2" to adjust the P value. The code is

"dds <-DESeqDataSetFromMatrix(countData=exprSet, colData=metadata, design=~sample,tidy=TRUE)

dds <- dds[rowSums(counts(dds))>1,]

dds <- DESeq(dds,parallel = T)".

And our subsequent analysis is based on the Padj value.

**Replies to Reviewer 1**

Reviewer #1: The present work describes the discovery of a novel prognostic biomarker for liver hepatocellular carcinoma patients. Using TCGA available RNA-seq and clinical patients’ data and applying bioinformatics approaches, authors unveil DYNC1|1 as the most significantly deregulated gene in a series of LIHC patients. I would like to address the following aspects related to this manuscript:

Major comments:

1. The main figures of the paper should represent the results described in the text. The figures selected do not have enough quality to be evaluated and the description in the legend is not well described. Please, add high quality figure that could be evaluated, a main title and a complete description of the plots and graphics represented, included the meaning of colour keys (colours should be correlating between figures) and symbols.

Answer:

Thank you for your suggestion, we have updated the clarity of the figures and changed the description of the figures.

2. Line 102-103. Please, include a table (in supplementary data) with the 20 most significant GO terms described in the text and 9 KEGG significant pathways with their score. Provide plots with more quality.

Answer:

Thank you for your suggestion, we have added the GO and KEGG data tables in the supplementary materials.

3. Line 119. Please provide a table (supplementary data) with the top ten genes obtained with the 11 different algorithms and their scores. Is only DYNC1|1 in the intersection of these algorithms? If not, include a ranking of the top ten (maximum) and their scores.

Answer:

Thank you for your suggestion. There is only DYNC1I1 in the intersection of TOP100, so we provide the scores of the top ten genes and the scores of all genes in the supplementary materials. The file name is " Cyto-Hubba _score".

4. It would be interesting if authors could provide a table with the interacting genes belonging to the same hub than DYNC1|1. It could complete the information and add some valuable insight about the possible functionality of this alteration in liver cancer (main text).

Answer:

Thank you for your suggestion. We have added a list of proteins that interact with DYNC1I1 in the main text.

5. Line 124. In the characterization of DYNC1|1 there is a lack of description. Authors showed in a figure the gene location and protein structure, but they did not add much detail in the text. Please, complete the description (family of the gene, which kind of protein codifies…), features of the structure and potential functionality of the gene (if it was previously described).

Answer:

Thanks for your suggestion, we have added a related description in the article.

6. Line 126. Authors described the significant difference of DYNC1|1 expression between LIHC and normal adjacent tissue, but they did not include the statistical value for this comparison in the text. The same was done when they compared male vs female in LIHC. Please, include all these statistics, and add in the “Methods” sections how these statistics were performed.

Answer:

Thank you for your suggestion. We have added the P value data to the main text, and described the use of the R package "DESeq2" to analyze the data in the method.

7. In line with the previous comments and comparisons performed, the individual comparisons “LIHC vs control” in male and women independently were missed, and due to the observation of the authors that the expression in male is significantly higher than in women, I was wondering whether DYNC1|1 would be still useful as a prognostic marker for women with LIHC, or if the overexpression of this gene will remain significant in this comparison. Please, include these comparisons in this section and their statistics.

Answer:

Thank you for your valuable comments. Our research found that the expression of DYNC1I1 in men is higher than that in women, and it can predict the prognosis very well in men, but not in women. We have added this part to the main text.

8. Line 133. Please, divide de survival rate also by gender in high and low DYNC1|1. Add the results in the text and the related graphics in supplementary information.

Answer:

Thank you for your comments. We have added this part to the main text.

9. Line 153 to line 180. This text seems like an introduction and summary of the authors’ work. Just a reminder that this is the “Discussion” section. I suggest beginning with the discussion in line 181, maybe including a different first sentence that complete the paragraph. The previous discarded text could be included in the short introduction of de manuscript.

Answer:

Thank you for your comments, we have made changes to this part of the content.

10. I should recommend authors to be more specific in some comments throughout the text because some of them like “its efficacy is still not optimistic” or “easy recurrence” do not sound very scientific. For avoiding being vague in the expression of this information, please provide previous statistics in related works supporting these comments.

Answer:

Thanks for your suggestions, we have modified these descriptions accordingly.

11. In the discussion there is some information missing. Please provide more detail about the results in other cancers with DYNC1|1 (the sense of the deregulation, possible role on these cancers, etc) and add a proper comparison with the results obtained in this work.

Answer:

Thank you for your suggestions. We have added and modified relevant content according to your suggestions.

12. Line 193. I am not totally sure what authors mean with the term “multi-cell cycle” and its regulation by DYNC1|1. Please, explain this concept.

Answer:

Thank you for your correction. Due to our negligence, we can see that DYNC1I1 regulates the cell cycle in KEGG, which we have corrected in the article.

Minor comments:

- Please, describe the abbreviations before including them in the text, i.e. like PPI (line 76).

Answer:

Thanks for your suggestion, we have revised the acronyms.

- Line 80: Please change the sentence: “We use MCC, DMNC…” and substitute for “We used 11 algorithms of the Cyto-Hubba software to screen out the hub gene: MCC, DMNC, MNC…” instead, for expressing the methods a clearer way.

Answer:

Thanks for your suggestion, we have made changes.

Reviewer #2: In this manuscript, the authors retrospectively analyzed the available TCGA data for LIHC to identify genes and pathways that are dysregulated in cancer compared to normal adjacent tissue. In their analyses, they identify DYNC1I1 as a hub gene in LIHC and a potential prognostic factor.

The analyses are well performed, however there are a few issues that should be solved before publications.

In general, I would highly recommend that they increase the font size of all the writing in the figures, most of which is currently not readable in the printed size.

Answer:

Thanks for your suggestion, we have updated the figures.

Some more details would be appreciated in the figure legends. For example: specify what are the blue dots in figure 1B and the red dots in figure 1D. The legend of figure 3 has not details at all, it only indicates the tools that were used. In Figure 4A is the red line indicating the position of the gene? Please specify these details.

Answer:

Thank you for your suggestions. We have added and modified relevant content according to your suggestions.

Cyto-Hubba identified DYNC1I1 as a hub gene. Was this the only gene at the intersection of the 11 lists? Please specify if this is the case.

Answer:

Yes, this is the only gene in the TOP100 among the 11 algorithms.

Since they show that DYNC1I1 expression is higher in male patients (Figure 4D), it would be interesting to stratify the survival by sex and determine whether the prognostic significance is different in male vs female patients.

Answer:

Thank you for your suggestion. DYNC1I1 has different effects on the survival of men and women, and we have added this content in the main text.

The first part of the discussion is more or less a repetition of the introduction and could be eliminated or incorporated in the introduction.

Answer:

Thanks for your suggestion, we revised the first part of the discussion.

In the discussion they mention that DYNC1I1 is an independent prognostic factor, however, this is not fully demonstrated, because no multivariate analysis of the survival data is performed. Without this analysis, they should remove “independent”.

Answer:

Thank you for your suggestion. We changed the "independent prognosis" to "prognosis".

Finally, I would suggest that the authors adhere the general practice of writing in the past tense, if possible.

Answer:

Thanks for your suggestion, we will ask native English speakers to help us modify the grammar of the article.
