# Supplementary material for: Bioinformatics analysis identifies DYNC1I1 as prognosis marker in male patients with liver hepatocellular carcinoma
Source: PLoS One. 2021 Oct 22;16(10):e0258797. doi: 10.1371/journal.pone.0258797 (PMC8535175; doi:10.1371/journal.pone.0258797)
Supplement: Attachment — Submitted filename: Response to Reviewers.doc [file pone.0258797.s012.doc]

**Response Letter**

Dear Editor-in-Chief,

Thank you very much for your letter and advice. We have revised the manuscript, and would like to re-submit it for your consideration. We have addressed the comments raised by the reviewers and editor, and the amendments are highlighted in red in the revised manuscript. The responses to the editor' and reviewers' comments are listed below this letter.

We hope that the revised version of the manuscript is now acceptable for publication in your journal.

Looking forward to hearing from you soon.

With kind regards,

Yours sincerely,

Yan Liu Dr.

Puren Hospital Affiliated to Wuhan University of Science and Technology, Wuhan, Hubei, 430081, China.

Tel/Fax: 027-86367776

Email: [liuyan@wust.edu.cn](mailto:liuyan@wust.edu.cn)

We would like to express our sincere thanks to the editor and reviewers for the constructive and positive comments.

**Replies to Journal Requirements**

Please review your reference list to ensure that it is complete and correct. If you have cited papers that have been retracted, please include the rationale for doing so in the manuscript text, or remove these references and replace them with relevant current references. Any changes to the reference list should be mentioned in the rebuttal letter that accompanies your revised manuscript. If you need to cite a retracted article, indicate the article’s retracted status in the References list and also include a citation and full reference for the retraction notice.

Answer: Thank you for your suggestions. We have revised and updated the references.

**Replies to Editor**

Please clarify package names and versions for all R packages used.

Answer: Thank you for your valuable comments. We updated the package name and version of all the R package.

Please also clarify parameters used in all software. If defaults were used please state “using default parameters” or similar in the text.

Answer: Thank you for your comment, we revised the relevant content.

You have clarified processing of the RNAseq data in the response to review: “use the code "dds <- dds[rowSums(counts(dds))>1,]" to delete low-expressed genes”. This information should be in the methods.

Answer: Thank you for your suggestions, we revised the relevant content.

Please ensure all methodology is covered, for instance the DAVID database is mentioned in the results section but not in the methods.

Answer: Thank you for your comment. We revised the relevant content.

Additionally, what is the source of the structural data in figure 4?

Answer: Thanks for your comment. Data was obtained from the online database Swiss-Model (<https://swissmodel.expasy.org/repository/uniprot/O14576?csm=55A6FF971E632DA0>), and we revised the relevant content in text.

Please add appropriate references for all software and databases used.

Answer: Thank you for your suggestions. We have added relevant references.

The STRING database contains multiple types of interaction from several sources, many of which are functional interactions rather than physical PPI eg co-expression data. Please detail the sources chosen and score threshold applied. If functional data are included in the network, please change the text accordingly as this is not a PPI network.

Answer: Thanks for your suggestion. Homo sapiens was selected for STRING analysis, and the detailed scoring value was added to the supplementary materials.

At line 213 please change “gender” to “sex” to clarify this is a biological classification.

Answer: Thank you for your comment, we have made changes to this part of the content.

**Replies to Reviewer 1**

Reviewer #1: The authors have completed the requests and added some valuable information and discussion about the different aspects pointed out during the first revision, improving considerably the quality of the manuscript. I am happy with the result of these changes. However, there is still some minor remaining aspects that I would like to point out before the manuscript will be ready for publication:

Minor comments:

1. Line 69. There are still some misspelling words. Please, substitute “considerd” for “considered” and “logFC <2.5 are significant” for “logFC >2.5 as significant”.

Answer: Thanks for your suggestions, we have made changes.

2. Lines 108-109. Please, include the information “using the R package “clusterprofiler” in Methods’ section, “Functional enrichment analysis” (line 73) where it corresponds. This information should be there and not in the Results’ section. Same happened with the sentence: “GO function annotation of the interacting proteins was performed using the R package” (line 170-171), move that information to the same section.

Answer: Thank you for your valuable comments. We have made corresponding modifications in the article.

3. Lines 142, 145, 148, 154. Please, substitute “than that” only for “than”.

Answer: Thank you for your suggestions. We changed the "than that" to "than".

4. Line 147. Write “and the results showed that…” instead of “and the resulted show that…”.

Answer: Thanks for your suggestion, we have made changes.

5. Lines 157-158. Please, insert here that “These results indicated that high expression of DYNC1|1…”.

Answer: Thanks for your suggestion, we have modified these descriptions accordingly.

6. Lines 186, 188, 189. Please, eliminate in the indicated lines the word “rate” after “recurrence”, “recurrence” and “survival” consecutively.

Answer: Thanks for your suggestions, we have made changes.

7. Lines 209, 211. Add “LIHC in male patients” instead “LIHC male patients”.

Answer: Thanks for your suggestions, we changed the "LIHC male patients" to "LIHC in male patients".

8. Line 212. Same here, add “LIHC for male patients”.

Answer: Thank you for your suggestion, we have made changes to this part of the content.

9. Line 213. “we will classify patients’ samples by gender…”.

Answer: Thanks for your suggestion, we have made changes.
